# A Multicentre Clinical Trial Protocol of Shuganjieyu Capsules Combined With Fluoxetine in the Treatment of Depression

**DOI:** 10.7759/cureus.84280

**Published:** 2025-05-17

**Authors:** Ji-tao Li, Xue-mei Liao, Lin-lin Zhu, Zhi-jian Yao, Zhi-fen Liu, Huan-zhong Liu, Xue-qin Song, Yun-ai Su, Tian-mei Si

**Affiliations:** 1 Psychopharmacology, National Clinical Research Center for Mental Disorders (Peking University Sixth Hospital/Institute of Mental Health) and the Key Laboratory of Mental Health, Ministry of Health (Peking University), Beijing, CHN; 2 Psychiatry, The Affiliated Brain Hospital of Nanjing Medical University, Nanjing, CHN; 3 Psychiatry, First Hospital of Shanxi Medical University, Taiyuan, CHN; 4 Psychiatry, Chaohu Hospital of Anhui Medical University, Hefei, CHN; 5 Psychiatry, First Affiliated Hospital of Zhengzhou University, Zhengzhou, CHN

**Keywords:** depression, efficacy, fluoxetine, safety, shuganjieyu capsules

## Abstract

Background

Depression is a global disease with high prevalence, recurrence, and disability rates. Currently, antidepressant chemical drug therapeutic regimens are the mainstay, but they have low recovery rates, and a significant portion of patients develop refractory depression. Consequently, traditional Chinese medicine (TCM) has emerged as a crucial alternative, with various studies investigating its potential in both standalone and adjunctive treatments for depression. Thus, this study aims to explore the efficacy and safety of an integrated TCM and Western medicine antidepressant therapeutic regimen, i.e., the combination of Shuganjieyu capsules and fluoxetine, in patients with moderate to severe depression (17-item Hamilton Rating Scale for Depression (HAMD-17) score ≥ 18 and Item 13 score ≥ 2) in the short- and long-term (8 weeks and 16 weeks, respectively).

Methods

The study will be divided into a basic study period (0-8 weeks) and an extended study period (9-24 weeks). The basic study period will employ a multicentre, randomized, double-blind, placebo-parallel controlled study, in which patients with moderate to severe depression will be selected and randomly assigned at a 1:1 ratio, with 80 patients in each group. The extended study period will utilize a multicentre, open-label observational study. During the basic study period, the experimental group will receive oral fluoxetine (20 mg/dose, once daily) and Shuganjieyu capsules (two capsules/dose, twice daily, morning and evening), whereas the control group will receive fluoxetine (20 mg/dose, once daily) and a Shuganjieyu capsule simulant (two capsules/dose, twice daily, morning and evening). The dosing regimen for the extended study period will be formulated on the basis of the condition of the subjects after 8 weeks of the basic study period. The primary efficacy endpoint will be the change in the HAMD-17, and the secondary efficacy endpoint will include the change in the Hamilton Anxiety Rating Scale (HAMA), Clinical Global Impression (CGI), Patient Health Questionnaire-15 (PHQ-15), Pittsburgh Sleep Quality Index (PSQI), Dimensional Anhedonia Rating Scale (DARS), Temporal Experience of Pleasure Scale (TEPS), and Quality of Life Enjoyment and Satisfaction Questionnaire-Short Form (Q-LES-Q-SF).

Discussion

This study aims to evaluate the efficacy and safety of the combination of TCM and Western medicine in the acute treatment of moderate to severe depression. Additionally, it seeks to explore the long-term efficacy (in terms of improving residual symptoms and preventing relapse) and safety of this combination therapy. The ultimate goal will be to develop an optimized treatment regimen for depression that combines traditional Chinese medicine and Western medicine, thereby offering more options for the clinical management of moderate to severe depression.

## Introduction

Depression is a highly disabling disorder worldwide that is characterized mainly by a significant and persistent depressed mood, manifested by symptoms such as low mood, diminished interest, and impaired cognitive functioning [1, 2]. Some patients may also present with somatization symptoms, such as insomnia or drowsiness, decreased appetite, fatigue, and hyposexuality. In severe cases, patients may engage in self-injurious or even suicidal behaviours [3], making it the third leading cause of death and disability [1]. According to the World Health Statistics 2023, it is estimated that 3.8% of the world’s population suffers from depression, including 5% of adults and 5.7% of elderly individuals aged 60 years and above [4]. Moreover, more than 10% of pregnant and postpartum women worldwide are affected by depression [5]. Research also predicts that, by 2030, depression may become the most prevalent disease worldwide [1].

Common treatments for depression currently include pharmacological and nonpharmacological therapies [6-10]. In pharmacological therapy, selective serotonin reuptake inhibitors (SSRIs) are among the most commonly used antidepressant drugs in clinical practice [11], and their mechanism of action primarily involves selectively inhibiting the reuptake of serotonin, thereby exerting antidepressant effects. The Sequenced Treatment Alternatives to Relieve Depression (STAR*D) study has shown that, after acute-phase treatment, there are differences in remission rates among different treatment regimens for patients with depression [12, 13]. The remission rate of the first-stage SSRI, citalopram, is only 36.8%. This study has revealed that 90% of patients with depression who responded to acute-phase treatment still present at least one residual symptom, and the most common residual symptoms include sleep disturbances and appetite/weight disturbances. Several studies have explored other augmentative treatment strategies, such as anti-inflammatory drugs and traditional Chinese medicine (TCM) therapy.

According to TCM, depression belongs to the category of “depression syndrome”, and its main clinical manifestations include depressed mood, emotional restlessness, fullness and stuffiness in the chest and subcostal regions, or ease of anger and crying [14]. TCM treatment emphasizes holistic regulation of the body and mind, achieving personalized treatment plans through multiple approaches, targets, and levels [15]. Integrative treatment combining TCM and Western medicine during the acute phase of depression can shorten the time for medication to take effect, rapidly relieve symptoms, synergistically enhance efficacy, and reduce the adverse effects of antidepressant chemical drugs. In the consolidation and maintenance phases, it can further stabilize symptoms, improve residual symptoms, prevent relapse, and prevent recurrence through holistic regulation [14].

Shuganjieyu capsule is the first traditional Chinese medicine approved by the National Medical Products Administration (NMPA) for the treatment of mild to moderate depression [16], and its main ingredients are extracts of *Hypericum perforatum *(HP) and *Eleutherococcus senticosus *(ES), which are known to effectively soothe the liver, relieve depression, strengthen the spleen, and calm the mind. Pharmacokinetic studies have shown that, when extracts of HP and ES are used in combination, they may synergistically exert antidepressant effects by increasing the plasma concentration of hypericin and shortening the time to reach the concentration of eleutheroside E [17]. The antidepressant mechanism of Shuganjieyu capsule has not been fully elucidated, but current research suggests that it may involve multi-target and multi-pathway regulatory effects. These include modulating the levels of monoamine neurotransmitters in the brain (such as serotonin, norepinephrine, and dopamine), inhibiting the release of inflammatory factors, and enhancing neuroplasticity, thereby exerting its antidepressant effects [18]. Previous studies have preliminarily observed the efficacy of combining Shuganjieyu capsules with antidepressant chemical drugs during the acute phase of depression. The results have indicated that the combination of antidepressant chemical drugs with Shuganjieyu capsules has a synergistic effect, especially on symptoms of depression, anxiety, and insomnia, compared with the antidepressant chemical drug monotherapy group [16, 19, 20]. However, research on the long-term efficacy and safety of their combined use remains limited. Given that depression is a chronic progressive disease, clarifying the long-term effectiveness and safety of integrative treatment combining Chinese and Western medicine will be highly important for clinical practice.

This study aims to combine the concept of whole-course management of depression and establish a basic study period (short-term) and an extended study period (long-term) in the project protocol. Through multidimensional scale assessment, this study will further explore the efficacy and safety of combining Shuganjieyu capsules with antidepressant chemical drugs for both short-term and long-term treatment of depression. Additionally, patient satisfaction with the treatment will be evaluated. The goal is to offer more suitable long-term treatment options for individuals with depression and enhance their overall quality of life.

## Materials and methods

Study design

This study will primarily aim to observe the therapeutic efficacy and safety of an integrated traditional Chinese medicine and Western medicine treatment regimen for depression. The study design will include a basic study period (randomized controlled trial (RCT), 8 weeks) and an extended study period (observational study, 16 weeks). The experimental group will be treated with Shuganjieyu capsules combined with fluoxetine for 24 weeks. In the control group, the medication regimen will be fluoxetine + placebo in the basic study period, and may contain Shuganjieyu capsules + fluoxetine (if 17-item Hamilton Rating Scale for Depression (HAMD-17) score reduction rate less than 50% on the end of week 8) or fluoxetine alone (if HAMD-17 score reduction rate more than 50% on the end of week 8) in the extended study period, so that the purpose of this study design will be to observe whether the early combination of Shuganjieyu capsules with fluoxetine as the basic treatment is more effective compared to fluoxetine alone in the depression patients with poor early efficacy.

**Figure 1:**
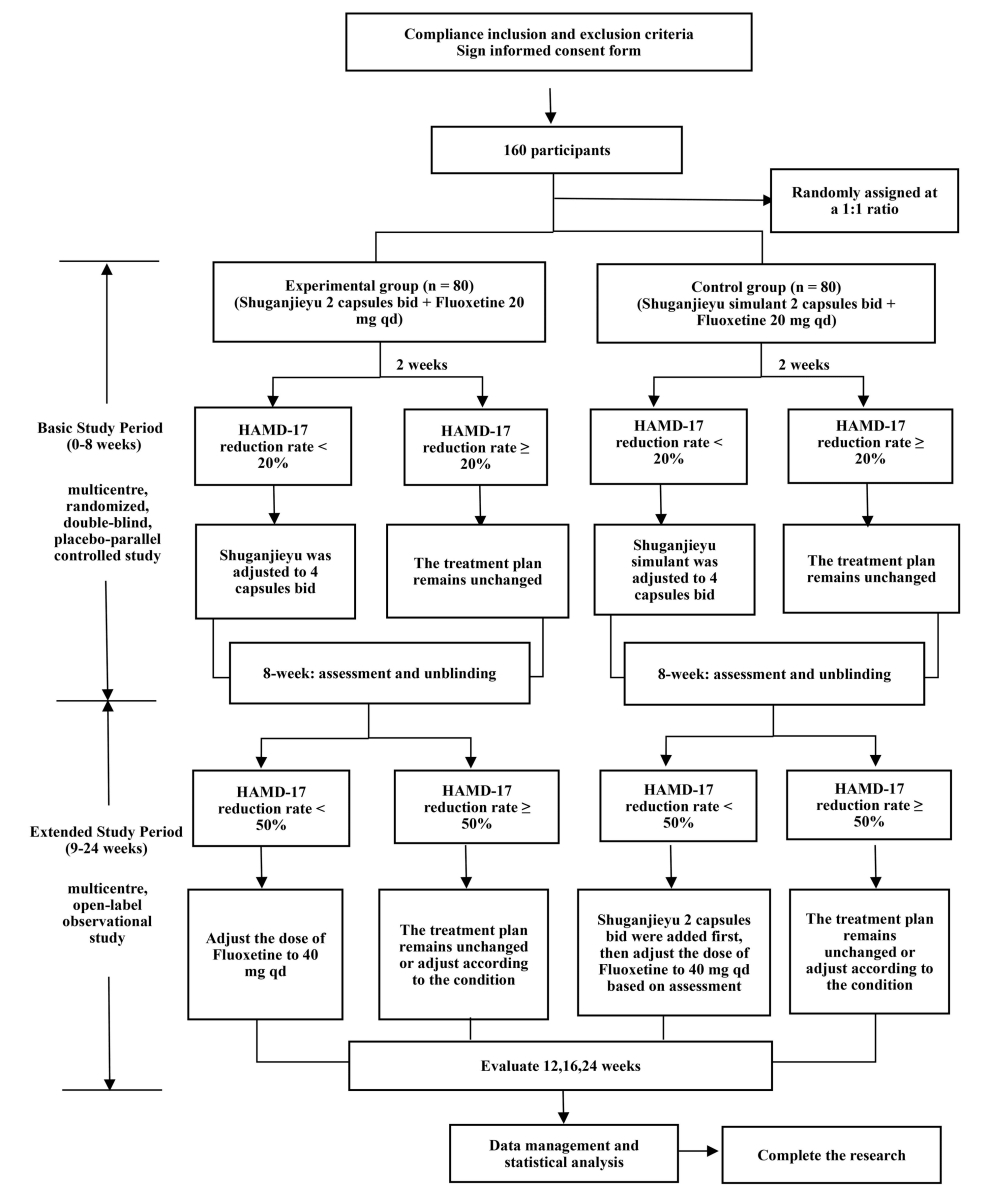
Flowchart of the study HAMD-17: 17-item Hamilton Rating Scale for Depression

The trial will consist of two phases: the basic study period and the extended study period. The overview of the study is shown in Fig. 1.

Basic Study Period (Weeks 0-8)

A multicentre, randomized, double-blind, placebo-controlled parallel design will be employed. During the basic study period, a stratified and block randomization method will be used. All participants will be randomly assigned at a 1:1 ratio to either the experimental group (Shuganjieyu capsules combined with fluoxetine) or the control group (Shuganjieyu capsule simulant combined with fluoxetine). The evaluation during the basic study period will be conducted via double-blind and single-simulation techniques; i.e., both the investigator and the participants will be required to remain “blinded” during the grouping and evaluation. Additionally, the preparation of the placebo used in the control group will ensure that it matches the experimental drug in sensory aspects (such as dosage form, shape, colour, texture, and odour). The placebo will not contain the active ingredients of the experimental drug. This design will minimize subjective errors and ensure the objectivity and reliability of the trial results.

Extended Study Period (Weeks 9-24)

A multicentre, open-label observational study will be conducted. After completing the 8-week treatment period in the basic study period, the investigator will adjust the dosing regimen according to the condition of the participants, either to continue maintaining the treatment regimen of the basic study period in the experimental/control group or to adjust the dosing regimen for the experimental group. The design of the extended study period aims to align with clinical practice.

Sample Size Calculation and Randomization and Blinding Methods

This study will be a superiority trial randomized 1:1 to the experimental group (Shuganjieyu capsules combined with fluoxetine) and the control group (Shuganjieyu capsule simulant combined with fluoxetine). The sample size will be calculated based on the following assumptions: the relative baseline change in the HAMD-17 score at 8 weeks is expected to be -13.3 for Shuganjieyu capsules combined with fluoxetine and -11 for the Shuganjieyu capsule simulant combined with fluoxetine. The difference in score reduction (δ) will be -2.3, the standard deviation (SD) will be 4.3, the two-sided type I error will be set at 0.05, and the statistical power will be 85%. Given a 20% dropout rate, each group will require 80 participants, resulting in a total of 160 participants.

In the basic study period of this trial, a stratified block randomization method will be adopted, with the study center as the stratification factor. Block randomization will be conducted within each center. The randomization list will be generated by the randomization statistician using SAS 9.4 statistical software (SAS Institute, Cary, USA) based on stratification factors and grouping ratios. Confidential parameters, including the number of blocks, block length, and random seed values, will be sealed in opaque envelopes along with the randomization list as the allocation schedule, which remains unopened during the trial. After completing the 8-week basic study period observation, investigators at each center will unblind the drug coding for individual patients.

Participant Selection

These criteria will standardize the study process, ensure its rigor, and protect the rights and interests of participants from an ethical perspective. The inclusion criteria for participants in this study will include the following: (1) meeting the diagnostic criteria for depression according to the Diagnostic and Statistical Manual of Mental Disorders (5th edition) (DSM-5); (2) aged 18-65 years (inclusive), regardless of sex; (3) HAMD-17 score ≥ 18 in the screening period, and Item 13 score ≥ 2; (4) negative pregnancy test; and (5) voluntary signing of the informed consent form and agreement to participate in all visits, examinations, and treatments according to the trial protocol. The exclusion criteria will include the following: (1) treatment-resistant depression (lack of clinical efficacy after adequate treatment with two or more different mechanisms of antidepressant drugs), depression caused by psychotropic drugs, depressive secondary to other mental illnesses, or somatic diseases at an active stage; (2) individuals meeting DSM-5 criteria for other mental disorders (such as schizophrenia spectrum and other psychotic disorders, bipolar and related disorders, anxiety disorders, obsessive-compulsive and related disorders, somatic symptoms and related disorders); (3) severe suicidal tendencies (HAMD-17 suicide score ≥ 3) or a tendency to harm others; (4) severe or unstable cardiovascular, cerebrovascular, liver, kidney, endocrine, digestive and blood diseases; liver function tests showing alanine aminotransferase (ALT), aspartate aminotransferase (AST) > 2 times the upper reference limit, or serum creatinine (SCr) > upper reference limit; patients with a history of hyperthyroidism, hypothyroidism, or other endocrine diseases currently in an active period; (5) patients who have undergone psychiatric surgery or electroconvulsive therapy in the past 3 months; those who have not responded to previous treatment with Shuganjieyu capsules or fluoxetine; those who have received or are currently receiving any other investigational drug therapy within 3 months before the trial; patients taking interfering drugs contraindicated with the investigational drug, or those who have received systemic antidepressant therapy within 4 weeks; patients with psychoactive substance abuse or dependence in the past 12 months; (6) patients with known or suspected allergies to *Hypericum perforatum*, *Eleutherococcus senticosus*, or fluoxetine; long-term use of caffeine and nicotine; and (7) other individuals who are deemed unsuitable for participation in this clinical trial by the researchers.

Participant treatment

Investigational Product

An overview of the schedule of assessments is shown in Table 1. During the basic study period (0-8 weeks), both the investigational drug Shuganjieyu capsules (0.36 g/capsule) and the placebo (simulant Shuganjieyu capsules) will be provided by Sichuan Jishengtang Pharmaceutical Co., Ltd. The basic drug fluoxetine (20 mg/capsule) is a commercially available drug that has undergone consistent evaluation.

**Table 1:** Assessment schedule *Laboratory examination: Routine blood tests, routine urine tests, liver function tests, and renal function tests HAMD-17, 17-item Hamilton Depression Rating Scale; HAMA, Hamilton Anxiety Rating Scale; CGI, Clinical Global Impression; PHQ-15, Patient Health Questionnaire-15; PSQI, Pittsburgh Sleep Quality Index; DARS, Dimensional Anhedonia Rating Scale; TEPS, Temporal Experience of Pleasure Scale; Q-LES-Q-SF, Quality of Life Enjoyment and Satisfaction Questionnaire-Short Form.

Phase	Basic study period				Extended study period		
Visit Items	Visit 1	Visit 2	Visit 3	Visit 4	Visit 5	Visit 6 (Telephone follow-up)	Visit 7
Visit time	-3 to 0 day (Screening)	2 weeks ±3 days	4 weeks ±3 days	8 weeks ±3 days	12 weeks ±3 days	16 weeks ±3 days	24 weeks ±3 days
Sign informed consent form	●						
Demographic information	●						
Inclusion and exclusion criteria	●						
Vital signs	●	●	●	●	●		●
Physical exam	●	●	●	●	●		●
Laboratory examination *	●			●			●
Electrocardiogram	●			●			●
HAMD-17	●	●	●	●	●	●	●
HAMA	●	●	●	●	●	●	●
CGI	●	●	●	●	●	●	●
PHQ-15	●	●	●	●	●	●	●
PSQI	●	●	●	●	●	●	●
DARS	●	●	●	●	●	●	●
TEPS	●	●	●	●	●	●	●
Q-LES-Q-SF	●			●			●
Drug administration plan	●	●	●	●	●	●	●
Concomitant medication	●	●	●	●	●	●	●
Adverse events	●	●	●	●	●	●	●

Before dispensing investigational drugs (Shuganjieyu capsules/placebo) to participants, they must be packed identically and without any distinguishing marks. The blinded investigational drugs for the basic study period, which have passed quality inspection, will be delivered to each clinical trial centre. Drug administrators designated by each centre will manage and distribute the drugs uniformly, maintaining relevant records. This process ensures compliance with the double-blind trial design requirements.

Dosage Regimen

During the basic study, the experimental group will receive oral fluoxetine (20 mg/dose, once daily) with Shuganjieyu capsules (two capsules/dose, twice daily, morning and evening). The control group will receive fluoxetine (20 mg/dose, once daily) with Shuganjieyu capsule simulant (two capsules/dose, twice daily, morning and evening). If the HAMD-17 total score reduction rate is less than 20% at the end of the second week, the dosage of Shuganjieyu capsule or simulant will be adjusted to four capsules/dose, twice daily, in the morning and in the evening.

After completing the 8-week basic study period and entering case report form (CRF) data, the investigators at each centre will unblind the drug codes corresponding to individual participants. On the basis of the participants’ condition after 8 weeks of the basic study period, an extended study period medication plan was formulated. For participants who enter the extended study period, if their HAMD-17 total score reduction rate is less than 50%, the fluoxetine dosage will be adjusted to 40 mg/dose once daily for the experimental group. For the control group, Shuganjieyu capsules (two capsules/dose, twice daily, morning and evening) will be added first, and further adjustments to the fluoxetine dosage (40 mg/dose, once daily) will be considered on the basis of changes in the participant’s condition. Participants will be prohibited from concurrently using other antidepressant medications during both the basic and extended study periods. During the basic study period, the use of other auxiliary treatments for depression will be prohibited, such as psychotherapeutic intervention and rehabilitation therapy, modified electroconvulsive therapy, transcranial magnetic stimulation, vagus nerve stimulation, deep brain stimulation, and other physical therapies. During the extended study period, the use of other auxiliary treatments for depression will be prohibited, including modified electroconvulsive therapy, transcranial magnetic stimulation, vagus nerve stimulation, and deep brain stimulation, but psychological interventions will be permitted.

Each clinical trial centre will perform unblinding only for participants who meet the specified criteria. Participants who do not complete the 8-week visit during the basic study period will remain blinded. Once all participants’ 24-week CRF data are fully entered and after any queries are addressed and no outstanding issues are observed, the data will be locked for final analysis.

Efficacy and safety indicators

Efficacy Indicators

The efficacy assessment indicators will include the following: (1) the primary efficacy endpoint will be the change in the HAMD-17; (2) the secondary efficacy endpoint will include the change in the Hamilton Anxiety Rating Scale (HAMA), Clinical Global Impression (CGI), Patient Health Questionnaire-15 (PHQ-15), Pittsburgh Sleep Quality Index (PSQI), Dimensional Anhedonia Rating Scale (DARS), Temporal Experience of Pleasure Scale (TEPS), and Quality of Life Enjoyment and Satisfaction Questionnaire-Short Form (Q-LES-Q-SF); the rate of patients with dose adjustments during the basic study period (8 weeks); the rate of relapse of depression symptoms at 12, 16, and 24 weeks of treatment in subjects who achieve clinical recovery/remission at 8 weeks of treatment; and the rate of medication changes/additions in participants who complete 8 weeks of treatment and enter the extended study period.

In this context, relapse will be defined as the recurrence of symptoms after achieving partial relief (effective, HAMD-17 reduction rate ≥ 50%) or clinical remission (complete symptom disappearance, HAMD-17 score ≤ 7) after the acute period of treatment [21].

Safety Indicators

The safety evaluation indicators will include the following: (1) vital signs: temperature, pulse, respiratory, and blood pressure; (2) routine blood parameters: red blood cell (RBC), haemoglobin (Hb), white blood cell (WBC), neutrophil percentage (NEUT%), and platelet (PLT); (3) routine urine parameters: urine protein (PRO), urine glucose (GLU), urine occult blood (BLD), and urine leucocyte (LEU); (4) liver function indicators: alanine aminotransferase (ALT), aspartate aminotransferase (AST), alkaline phosphatase (ALP), total bilirubin (TBIL), and gamma-glutamyl transferase (GGT); (5) renal function indicators: blood urea nitrogen (BUN), serum creatinine (Scr), uric acid (UA), and estimated glomerular filtration rate (e-GFR); (6) electrocardiogram examination: standard 12-lead electrocardiogram; and (7) adverse events: all adverse events occurring during the whole trial will be recorded at any time. The subsequent safety analysis includes an analysis of the potential correlation between adverse events and the investigational drugs. When adverse events occur, the safety of the subjects will be given first priority. Depending on the nature and severity of the adverse events, different treatments will be implemented, such as termination of the clinical trial, adjustment of drug dosage, and symptomatic treatment.

Quality control

This study was approved by the Ethics Committee of Peking University Sixth Hospital (approval NO. 2022-3) and registered at Clinicaltrials.gov (identification number: NCT05361330; Registered 4 May 2022, https://clinicaltrials.gov/study/NCT05361330). Investigators and their affiliated medical institutions will be required to follow the protocol approved by the Ethics Committee and the Clinical Trials Act during clinical trials to ensure the legality and compliance of the trial process. Additionally, during the course of the project, any deviations from the approved trial protocol will be thoroughly documented, explained, and properly archived by the trial institution and the project undertaker.

Given that this study is a multicentre clinical trial, the primary efficacy assessment will be based on scale evaluations. To ensure consistency in scale assessments across multiple centres, systematic training on the study protocol and scales will be conducted before project initiation. Standard operating procedures (SOPs) will be established on the basis of relevant study procedures and patient management.

In addition, to ensure the accuracy and reliability of the trial data, all the clinical examination laboratories of the medical institutions involved in the clinical trials will establish high-standard quality management and operation. For non-laboratory indicators, such as physical examinations, temperature measurements, and blood pressure measurements, each research unit will select recognized and quality-controlled measurement methods and instruments to ensure consistency when conducting examinations across different centres and personnel.

The project will have clinical research associates who have received professional training, with adequate scientific and clinical knowledge, as well as the required qualifications. Moreover, the project team will formulate a detailed monitoring plan and SOP to ensure the unified monitoring process and standards of clinical research associates. After the trial started, clinical research associates will conduct regular onsite monitoring visits and keep relevant records at the trial institution to ensure the accuracy and completeness of the trial records and reports. Moreover, they will actively monitor and check the implementation of the clinical trial protocol to ensure that the trial strictly follows relevant norms and regulations, guaranteeing its accordance with scientific principles, research rigor, and compliance.

Data collection and management

Data Collection

This study will focus primarily on scale assessments, with data collection to include demographic information, mood scales, somatic symptom scales, and quality of life scales.

Demographic information: Demographic information will be collected from the demographic characteristics (e.g., age, sex), baseline data, and previous treatments of the participants in each group to evaluate the baseline balance between the two groups.

Mood indicators: The HAMD-17, HAMA, TEPS, and DARS will be used to assess the participants’ depression, anxiety, and daily experiences of pleasure. These scales will provide a comprehensive understanding of various aspects, from emotional states to physiological symptoms.

Somatic symptom indicators: The PHQ-15 and PSQI will be used to assess the participants’ symptoms of somatic discomfort and sleep quality. These two scales will provide validated measures for the medical field and help provide a comprehensive understanding of patients’ somatic symptoms and sleep status.

Overall quality of life indicators: The CGI and the Q-LES-QSF will be used for assessment. The CGI will focus on the participants’ overall assessment of their clinical impressions and treatment satisfaction. The Q-LES-QSF will evaluate participants’ happiness and satisfaction across different aspects of daily life.

Data Entry and Management

In this study, an electronic data capture (EDC) database will be established, and all the data will be collected in the form of electronic case report forms (eCRFs). EDC will be employed in this multicenter trial to ensure data consistency across study sites and enhance traceability, both of which are critical for maintaining compliance with Good Clinical Practice (GCP) guidelines (ICH-GCP E6(R3), Sections 2.10 & 5.5) [22]. EDC systems minimize transcription errors, enable real-time data validation, and provide an audit trail for regulatory inspections, thereby improving overall data integrity and reliability. Within this trial, the investigators will meticulously record various information, including informed consent, enrolment time, use of investigational drugs, vital signs, relevant physical examinations, laboratory tests, scale assessments, concomitant medications, and adverse events. The clinical research coordinators (CRCs) will be responsible for accurately, promptly, completely, and consistently entering the raw data into the system, ensuring traceability for each data point.

The data manager will need to be trained in data management-related SOPs and familiarized with data management-related regulations and guidelines before participating in project work. Data management will include data validation and cleaning. The purpose of data validation will be to ensure data accuracy and completeness. The data manager will formulate a detailed and complete data validation plan (DVP) on the basis of the protocol and CRF. These plans will include automated logic checks, SAS program checks, manual checks by medical/data administrators, and source data verification (SDV). The clinical research associate will be responsible for the SDV, which will validate the consistency of the eCRF data with the source data by reviewing the original visit records. For laboratory and other external data, the clinical research associate will perform 100% of the source data verification.

Statistical analysis

A statistical analysis plan (SAP) will be written on the basis of the study protocol. The SAP will provide further detailed descriptions of the statistical analysis content and methods.

The statistical analysis datasets will include the full analysis set (FAS), the per-protocol set (PPS), and the safety set (SS). All statistical tests will be conducted as two-sided tests. Descriptive statistics, such as the mean, median, standard deviation, maximum, minimum, Q1, and Q3, will be used to summarize the data. All statistical analyses will be performed via SAS version 9.4 or above to ensure the accuracy and reliability of the results. Missing items in study scales will be primarily addressed using mixed-effects model repeated measures (MMRM), observed case (OC), or last observation carried forward (LOCF) imputation methods.

The overall statistical analysis will include participant distribution, demographic and baseline characteristics, efficacy analysis, and safety analysis. With respect to participant distribution, we will summarize the number of screened participants, those randomized into the study, and those who completed the trial. Additionally, we will conduct in-depth analyses of dropouts and their reasons for discontinuation. Treatment compliance will be assessed by pill counts and patient diary using the formula: Compliance (%) = (actual dose (tablets)/theoretical dose (tablets)) × 100.

In terms of demographic and baseline characteristics, descriptive statistics will be used to summarize relevant information about the participants in each group, including their past treatment history. For efficacy analysis, the change in HAMD-17 score at week 8 relative to baseline for the primary efficacy indicator will be statistically analyzed via MMRM and analysis of covariance (ANCOVA). All secondary efficacy indicators will be analyzed via a group t-test. For safety analysis, the Medical Dictionary for Regulatory Activities (MedDRA) will be used to code adverse events, and the number of cases, number of participants, and incidences of all adverse events, adverse events related to the test drug, and serious adverse events will be calculated separately for each group. Additionally, clinical judgement results for laboratory test outcomes before and after treatment will be summarized via contingency tables.

## Results

This article is a study protocol about the efficacy and safety of an integrated traditional Chinese medicine (TCM) and Western medicine antidepressant therapeutic regimen, i.e., the combination of Shuganjieyu capsules and fluoxetine, in patients with moderate to severe depression in the short and long term. This research is ongoing, and no results are available so far.

## Discussion

Depression is a serious mental disorder that significantly impacts public health. Currently, antidepressants remain the mainstay treatment for depression. However, commonly used antidepressants have shortcomings, such as a late onset of action and a low remission rate. Meta-analyses have shown that the clinical remission rate of patients receiving antidepressant chemical drugs is less than 30% [23]. Additionally, due to numerous adverse reactions, especially in the early stage of treatment, patient adherence to medication is severely affected [24]. With the development of integrated traditional Chinese and Western medicine, treating depression from a holistic viewpoint using this approach has been advocated. Short-term studies have suggested [14, 24] that integrated traditional Chinese and Western medicine may improve the efficacy of acute-phase depression treatment. However, currently, there is no evidence-based support for the long-term use of integrative treatment for depression. Further studies are needed to establish more effective integrated traditional Chinese and Western medicines for depression prevention.

This study will be conducted from the perspective of integrated traditional Chinese and Western medicine. For the treatment regimen, the classic SSRI fluoxetine will be chosen as the Western medicine. Although new antidepressants are constantly on the market, there is a minimal discernible difference in antidepressant efficacy between fluoxetine and newer agents. Fluoxetine has good efficacy for all types of depression and is recommended by the guidelines as the first-line treatment for depression [11, 25]. For traditional Chinese medicine, Shuganjieyu capsules will be selected. Shuganjieyu capsule is the first Chinese patent medicine approved for the treatment of mild-to-moderate depression in China. Since its launch in 2009, it has been widely used to treat mild-to-moderate depression and depression associated with other medical conditions, and substantial clinical evidence has accumulated [16]. The observation period for this study will be 24 weeks, covering both the acute and continuation phases of depression treatment. The study design will combine randomized double-blind and open-label observational studies to comprehensively assess the short- and long-term benefits of the integrated traditional Chinese and Western medicine treatment regimens. During the open-label phase, adjustments to medication regimens and dosages will be allowed on the basis of individual patient conditions, making the study more relevant to real-world clinical practice. The double-blind design will help eliminate subjective factors, making the results more objective and credible. The open-label observational study will allow for more flexible individualized adjustments to treatment plans on the basis of each participant’s situation, better aligning with clinical reality. The overall assessment criteria will cover the multidimensional symptomatic manifestations of depression, such as mood indicators, somatic indicators, and social functioning, which will help to more comprehensively explore the changes in the overall physical and mental conditions of patients in the course of treatment. This innovative multidimensional evaluation system will provide more detailed and comprehensive data, providing richer reference information for the comprehensive assessment of depression treatment. On the basis of the study design, this study aims to further validate the efficacy of Shuganjieyu capsules combined with fluoxetine in the short term (acute phase) for overall treatment effectiveness in patients with moderate to severe depression. Additionally, it aims to assess the efficacy of managing residual symptoms and preventing relapse in the long term (continuation phase). Throughout the entire study period, the impact on social functioning will also be observed.

In summary, this study will provide richer scientific evidence for the treatment of integrated traditional Chinese and Western medicine. This study aims to enhance the rational application of depression treatment in clinical practice, improve the condition and quality of life of depressed patients, and reduce the burden on families and society.

Limitations

This study has several limitations. First, it will be conducted only in China, although including the Han and ethnic minorities populations, and the sample size is relatively small; thus, the information it suggests needs to be further verified in large-sample or real-world studies. Additionally, whether there are racial and cultural differences remains unclear, so it should be investigated in a multinational, multicenter study in the future. Second, the assessment indicators used in this study are mainly scale indicators, and objective assessments, such as brain imaging, are lacking. Third, whether the efficacy and safety advantages of combining Shuganjieyu capsules with fluoxetine can be replicated in other integrated traditional Chinese and Western medicine regimens requires further controlled studies. Additionally, the sample of this study does not include patients with suicidal tendencies, limiting the generalizability of our findings to these high-risk populations. Additional studies are needed to validate these results across the full spectrum of depressive disorders. Finally, the current study will initially explore the mechanism of action of Chinese patent medicines, such as Shuganjieyu capsules, in the treatment of depression, but if both Chinese patent medicines and antidepressant chemical drugs are used, whether the substance basis and mechanism of action will change also needs to be further studied. Therefore, the research about drug-drug interactions and pharmacogenomic considerations should be explored in the future, and the mechanism of traditional Chinese medicine in improving depression should be further clarified in future research, including biomarkers or neurobiological targets grounded in monoamine and neurotrophic theory.

## Conclusions

This article is a study protocol about the efficacy and safety of an integrated traditional Chinese medicine (TCM) and Western medicine antidepressant therapeutic regimen, i.e., the combination of Shuganjieyu capsules and fluoxetine, in patients with moderate to severe depression in the short and long term. The research is ongoing, and no results are available so far. Therefore, there is no corresponding conclusion at present.
